# Single-Frame Infrared Image Non-Uniformity Correction Based on Wavelet Domain Noise Separation

**DOI:** 10.3390/s23208424

**Published:** 2023-10-12

**Authors:** Mingqing Li, Yuqing Wang, Haijiang Sun

**Affiliations:** 1 Changchun Institute of Optics, Fine Mechanics and Physics, Chinese Academy of Sciences, Changchun 130033, China; limingqing21@mails.ucas.ac.cn (M.L.); sunhaijiang@126.com (H.S.); 2 University of Chinese Academy of Sciences, Beijing 100049, China

**Keywords:** infrared image, non-uniformity correction, wavelet transform, cluster analysis, surface fitting

## Abstract

In the context of non-uniformity correction (NUC) within infrared imaging systems, current methods frequently concentrate solely on high-frequency stripe non-uniformity noise, neglecting the impact of global low-frequency non-uniformity on image quality, and are susceptible to ghosting artifacts from neighboring frames. In response to such challenges, we propose a method for the correction of non-uniformity in single-frame infrared images based on noise separation in the wavelet domain. More specifically, we commence by decomposing the noisy image into distinct frequency components through wavelet transformation. Subsequently, we employ a clustering algorithm to extract high-frequency noise from the vertical components within the wavelet domain, concurrently employing a method of surface fitting to capture low-frequency noise from the approximate components within the wavelet domain. Ultimately, the restored image is obtained by subtracting the combined noise components. The experimental results demonstrate that the proposed method, when applied to simulated noisy images, achieves the optimal levels among seven compared methods in terms of MSE, PSNR, and SSIM metrics. After correction on three sets of real-world test image sequences, the average non-uniformity index is reduced by 75.54%. Moreover, our method does not impose significant computational overhead in the elimination of superimposed noise, which is particularly suitable for applications necessitating stringent requirements in both image quality and processing speed.

## 1. Introduction

Infrared imaging systems possess robust diffraction penetration capabilities and operate effectively under all weather conditions. They have been widely employed in domains such as fire monitoring, night vision, target reconnaissance, remote sensing, and guidance [1]. In particular, the demand for civilian applications has been on the rise, encompassing areas such as enhanced perception for autonomous driving and short-wave infrared in vivo fluorescence imaging for medical purposes [2,3,4]. Nevertheless, infrared focal plane detectors inevitably encounter non-uniformity noise issues, manifested in images as striped artifacts and localized luminance non-uniformities. The principal factors contributing to such noise stem from the inherent unevenness in individual pixel responses, inconsistencies in signal readout circuit amplifier gains [5], and radiation effects stemming from detector window thermal sources [6]. Given the current state of manufacturing technology, overcoming non-uniformity noise entirely at the hardware level within a short timeframe remains unfeasible. Therefore, non-uniformity correction (NUC) must be performed in engineering applications where high imaging quality is required.

The purpose of NUC is to restore a clear image from a noisy image. This is a classic ill-posed problem. To address this challenging issue, numerous calibration-based NUC (CBNUC) methods have been proposed [7], including two-point correction, multi-point correction, and curve fitting correction. Additionally, scene-based NUC (SBNUC) methods have been introduced to estimate fixed pattern noise in real time based on the correlations between image sequences. These include temporal high-pass filtering methods [8,9,10], constant statistical methods [11,12,13,14], neural network methods [15,16], and image registration methods [17]. The aforementioned algorithms can yield satisfactory results in environments with limited temperature variations and ample field motion. However, they falter in complex working conditions, such as aerospace and medical imaging scenarios, where one must contend with uncertain external environmental changes and extended gaze exposure. This precludes the attainment of temperature-adaptive single-frame image NUC.

In pursuit of a more effective means to eradicate non-uniformity noise from single-frame images, researchers have endeavored to devise various correction methodologies capable of directly estimating noise from degraded images. Given the high-frequency attributes of stripe non-uniformity noise, frequency decomposition [18] techniques are typically employed as a preprocessing step for stripe removal. Subsequently, noise removal is achieved through corresponding noise suppression schemes [19]. In recent years, the most prevalent approach has been the direction-sensitive enhancement of stripe noise capture, predicated on one-dimensional guided filtering [20,21]. Furthermore, certain newly introduced models, such as the wavelet principal component analysis [22] and weighted least squares models [23], have been employed to refine noise estimation, facilitating single-frame image NUC. Nevertheless, models based on high-frequency noise characteristics have failed to account for non-uniformity noise induced by thermal radiation. Thermal radiation non-uniformity noise exhibits smooth low-frequency characteristics. Surface fitting and optimal estimation can effectively remove such noise [24,25,26]. However, these fitting-based estimation methods lack reasonable prior information to guide noise structure and often resort to the direct solving of optimal estimates through loss function construction. This approach necessitates iterative parameter adjustments, which not only significantly augment algorithm runtime but may also lead to parameter entrapment in local optima or divergence. With the evolution of deep learning, numerous effective NUC networks have been proposed [27,28]. However, owing to the scarcity of genuine paired training samples, these learning-based approaches have struggled to attain robust generalization performance in real-world NUC challenges.

Despite the significant progress achieved in the field of NUC, the aforementioned algorithms fail to address a multitude of complex issues, including environmental temperature variations, insufficient scene motion, low-frequency non-uniformity, and high-frequency non-uniformity. In order to concurrently tackle these diverse challenges, this paper combines the advantages of multi-scale wavelet transform and Bezier surface fitting to propose a single-frame image NUC method based on wavelet domain noise separation. This method defines the non-uniformity noise of the image as a combination of stripe non-uniformity noise and thermal radiation non-uniformity noise. To address the stripe non-uniformity noise, a clustering algorithm is employed for decomposition within the vertical component of the wavelet domain. For the thermal radiation non-uniformity noise, a Bezier surface fitting is applied to the approximated component in the wavelet domain. The proposed method provides corrected output results for single-frame images without relying on extensive camera motion within the field of view, thus avoiding the occurrence of ghosting artifacts.

The contributions of this paper are summarized as follows:

- This paper introduces a noise model that accounts for the superposition of stripe non-uniformity noise and thermal radiation non-uniformity noise. In contrast to previous correction models, this model can effectively address the intricacies of non-uniformity noise present in real-world scenarios;
- In order to remove high-frequency noise, this paper designs a scheme based on cluster analysis in the wavelet domain for strip non-uniformity noise. This scheme adaptively generates clusters according to the varying strengths of noise components. By categorizing the wavelet domain’s vertical components into noise information and detail information, this approach effectively removes stripe noise while preserving the utmost level of image detail;
- In order to remove low-frequency noise, this paper designs a scheme based on fitting the approximated components in the wavelet domain for thermal radiation non-uniformity noise. This approach capitalizes on the alignment between low-frequency components in the wavelet domain and the thermal radiation noise. By employing Bezier surface fitting, the scheme reconstructs a smoothed representation of the thermal radiation noise, thereby enhancing the overall uniformity of the corrected image.

The remainder of this paper is organized as follows: In Section 2, related works in this field are introduced. In Section 3, we provide a detailed derivation of the NUC method proposed in this paper. Section 4 presents a comparative experiment involving both simulated and real image sequences. Section 5 discusses the results of the study and points out the limitations of the proposed algorithm. Finally, Section 6 summarizes the article.

## 2. Related Work

Depending on the necessity of referencing sources such as blackbody radiation experiments, algorithms for NUC can be categorized into calibration-based NUC and scene-based NUC. Furthermore, scene-based NUC methods can be further subdivided into multi-frame scene-based NUC and single-frame scene-based NUC.

### 2.1. Calibration-Based Non-Uniformity Correction

Extensive research has been conducted in the field of calibration correction theory, yielding notable results. This approach is characterized by its simplicity and ease of engineering implementation. Common techniques encompass single-point calibration, two-point calibration, and multi-point calibration. Presently, the primary focus lies in the domain of temperature-adaptive models. In 2019, Chang et al. [7] proposed a two-point non-uniformity correction algorithm based on a single-reference image, effectively reducing the cost and complexity associated with traditional two-point algorithms. In the year 2022, Lin et al. [29] introduced a novel no-shutter correction scheme based on multi-variate polynomial correction, building upon the foundation of two-point calibration. This innovative algorithm leverages temporal variations in measurements of multiple camera temperatures to mitigate parameter perturbations induced by heating. Furthermore, based on this calibration model, a multitude of real-time non-uniformity correction systems implemented in hardware have been devised [30]. However, these methods, reliant on stable or slow-changing working environments, still fail to prevent parameter drift after prolonged continuous operation, necessitating recalibration that interrupts the normal functioning of the equipment.

### 2.2. Multi-Frame Scene-Based Non-Uniformity Correction

In pursuit of mitigating the constraints of CBNUC methods, the initial proposition was a scene-based non-uniformity correction approach reliant on the inherent relationships within multiple frame images. Notable methods within this category encompass temporal high-pass filtering algorithms, neural network algorithms, constant statistical algorithms, and image registration-based algorithms. Qian et al. [8] enhanced the classical temporal high-pass filtering NUC method through thresholding, rendering the management of high-frequency noise components controllable. Building upon this foundation, Zuo [9] and Zhang et al. [10] introduced bilateral filtering, guided filtering, and adaptive time constants, achieving more precise detail extraction and improved ghosting suppression capabilities. Lai et al. [15], building on neural network algorithms, introduced a novel adaptive filter based on the variable-step normalized mean squared error, expediting algorithm convergence. Rong et al. [16] proposed employing motion detection to determine the necessity of recalibrating coefficients, effectively overcoming the blurriness issue inherent in neural network approaches, and successfully deploying this algorithm on FPGA-based hardware platforms. Constant statistical methods assume that the average statistical data for each pixel should remain constant over an extended period, necessitating ample target motion within the image sequence. Harris et al. [11] suggested using thresholds to guide parameter updates in constant statistical algorithms, offering a straightforward and universally applicable strategy for scene-based correction. Various novel statistical strategies [13,14] have been introduced to suppress ghosting in static scenes during the statistical process. Zhang et al. [12] argued that statistical data for temporal domain signals may not be entirely consistent across individual pixels but remain constant within a local region. By leveraging local constant statistics, more effective noise removal can be achieved. Additionally, several frame registration-based methods [17] have been proposed; however, they are only suitable for minor non-uniformity noise and may introduce significant registration errors in cases of substantial noise.

These aforementioned multi-frame image-based scene non-uniformity correction algorithms are applicable solely within work environments characterized by ample field motion and are ineffective in eliminating non-uniformity noise in long-duration static image sequences.

### 2.3. Single-Frame Scene-Based Non-Uniformity Correction

In comparison to multi-frame scene-based non-uniformity correction algorithms, single-frame image non-uniformity correction algorithms prove more effective in avoiding ghosting and blurring issues, as they do not rely on guidance from adjacent frame correlations. Noise suppression schemes following frequency decomposition effectively segregate relevant image information from noise interference, commonly employed in various noise removal tasks, including non-uniformity correction. For instance, Liu et al. [19] introduced an image dehazing algorithm based on a unified variational model. This method decomposes the blurred image into a smooth illuminance component and a detail-rich reflection component, permitting independent dehazing and contrast enhancement treatments for each component. Yan et al. [18] devised a dual-module single-frame image denoising algorithm, enhancing high-frequency texture information and suppressing low-frequency glare and haze in a grayscale enhancement module to improve image quality. Zhang-T et al. [22] proposed a non-uniformity correction algorithm based on wavelet transformation, disassembling the degraded image into information of varying frequencies in the wavelet domain, subsequently utilizing the primary component in the vertical high-frequency domain for non-uniformity noise correction. Li et al. [20] employed a one-dimensional guided filter to extract high-frequency non-uniformity noise, followed by linear regression fitting to obtain correction coefficients for each pixel in high-resolution image processing, resulting in significant speed enhancements. Cao et al. [21] and Li et al. [23] similarly utilized guided filtering to acquire coarse noise images. However, Cao et al. separated stripe noise by exploiting local linear relationships between infrared data and stripe noise, while Li et al. improved stripe noise estimation using local weighted ridge regression.

The aforementioned algorithms predominantly target high-frequency non-uniformity noise removal, exhibiting suboptimal performance on global low-frequency thermal radiation non-uniformity noise. Limited research has been conducted on low-frequency non-uniformity correction caused by thermal radiation. To effectively leverage the global smooth property and local non-uniform characteristics of this noise, Liu et al. [24] adopted a TV regularization model to estimate thermal radiation noise, mitigating its impact on image details through weighted least squares filtering. Shi et al. [25] amalgamated spatial surface fitting regularization and multi-scale iterative estimation, presenting a Chebyshev polynomial rapid surface fitting-based multi-scale thermal radiation effect correction method, enhancing the robustness of optimal estimates effectively. Hong et al. [26] introduced a progressive thermal radiation noise correction method based on Bezier surface fitting. Compared to traditional single-degradation model approaches, the progressive correction model accurately fits noise deviation fields but incurs increased algorithmic time consumption.

With the rapid advancement of deep learning technology, numerous effective single-frame image non-uniformity correction networks have been proposed. For instance, Kuang et al. [27] introduced a dual-network architecture comprising denoising networks and conditional discriminators, utilizing the discriminator network to make the denoised output image resemble the target more closely. Chang et al. [28] incorporated wavelet transformation into convolutional neural networks, using wavelets to perceive the internal directionality of stripes as prior guidance for CNN learning, resulting in more efficient residual noise elimination. However, due to the scarcity of real paired training samples, especially in cases involving complex superimposed noise, these learning-based methods struggle to achieve strong generalization performance in real-world non-uniformity correction problems.

As previously elucidated, existing non-uniformity correction algorithms struggle to address a multitude of complex issues, including varying environmental temperatures, insufficient scene motion, and the amalgamation of low-frequency and high-frequency non-uniformities. To simultaneously overcome these challenges, we propose a single-frame image non-uniformity correction algorithm based on wavelet domain noise separation. This method defines the image’s non-uniformity noise as a combination of stripe non-uniformity noise and thermal radiation non-uniformity noise. For addressing stripe non-uniformity noise, we employ a clustering algorithm within the vertical components of the wavelet domain for noise separation. Concerning thermal radiation non-uniformity noise, separation is achieved through Bezier surface fitting of the wavelet domain’s approximation components. In this manner, the algorithm achieves simultaneous removal of both high-frequency and low-frequency non-uniformity noise from a single-frame image.

## 3. Proposed Method

### 3.1. Non-Uniformity Model

In general, the response curve of infrared focal plane array detector pixels is approximated using a linear model [1,5], and the mathematical formula for this model is represented as follows:

(1) T(i,j)=G(i,j) · f(i,j)+O(i,j)

where T(i,j) is the degraded image with non-uniformity noise, f(i,j) is the potentially clear image, G(i,j) represents the gain of pixel response, and O(i,j) denotes the offset in pixel response. It has been demonstrated by previous scholars [1,29] that in the majority of infrared focal plane array detectors, the dominant factor contributing to non-uniformity is bias non-uniformity. In fact, assuming that sensor parameters remain stable over a short period when detector pixels receive input signals of varying intensities, bias non-uniformity remains constant while only gain non-uniformity undergoes variation. The overall system’s non-uniformity can be regarded as fluctuating along a fixed baseline of bias non-uniformity as the reference. Furthermore, gain non-uniformity does not affect bias non-uniformity. Consequently, we can simplify the system’s non-uniformity noise to bias non-uniformity noise. The experimental section in Section 4 also validates that our simplification does not compromise the effectiveness of non-uniformity correction.

This paper establishes a degraded image model encompassing high-frequency stripe and low-frequency thermal radiation noise. The mathematical formulation of this degradation model is expressed as follows:

(2) T(i,j)=f(i,j)+s(i,j)+b(i,j)+n(i,j)

where T is the degraded image with non-uniformity noise, f is the potentially clear image, s is the stripe, b is the thermal radiation noise, and n represents the system noise.

**Figure 1:**
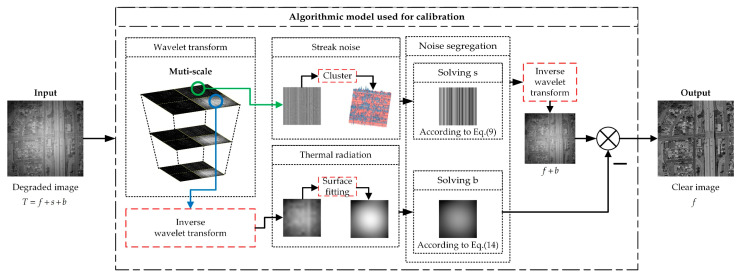
Framework of our proposed method.

The objective of NUC is to estimate the two types of non-uniformity noise, i.e., s and b, from the degraded image T, and subsequently eliminate the corresponding noise components to attain a clear image. Given the evident structural disparities between stripe and thermal radiation noise, it becomes imperative to decompose the non-uniformity noise into two distinct entities for separate rectification endeavors. In the approach proposed in this paper, a wavelet domain clustering analysis algorithm is employed for segregating stripe, whereas thermal radiation noise is separated through a wavelet domain guided spatial domain Bézier surface fitting technique. This process, assuming a negligible presence of system noise, is elaborated in its entirety, as depicted in Figure 1.

### 3.2. Wavelet Transform of Images

**Figure 2:**
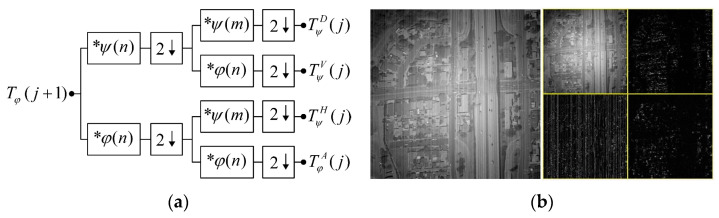
Two-dimensional fast wavelet transform: (**a**) decomposition filter bank; (**b**) 1—scale wavelet transform results.

Wavelet theory forms the fundamental basis of multi-resolution signal processing and analysis. Employing wavelet decomposition in the domain of image processing allows for the effective highlighting of localized characteristics within the problem, thus facilitating a more focused examination of specific regions pertaining to the task at hand. In digital image processing, the two-dimensional fast wavelet transform is commonly implemented using digital filters and down-samplers, as depicted in Figure 2.

In the diagram, $T_{\varphi }(j+1)$ represents the wavelet transform’s approximation coefficients. Here, j signifies the number of scale decomposition levels. The initial decomposition typically employs the original high-resolution image as input. ψ(n) denotes the scale coefficient, φ(n) stands for the wavelet coefficient, and m and n serve as variables in the convolution process, essentially representing the image’s resolution. ↓ represents the down-sampling process, and ∗ signifies convolution operations. Upon a single wavelet transform, an image yields an approximate image with low-frequency components and three detailed images containing high-frequency components in the horizontal, vertical, and diagonal directions, respectively. Following j iterations of wavelet transform, the input image can be represented as

(3) $$T_{\varphi }^{}=\left\{\;T_{\varphi }^{A}(j)\;,\;T_{\psi }^{H}(j)\;,\;T_{\psi }^{V}(j)\;,\;T_{\psi }^{D}(j),\;T_{\psi }^{H}(j-1)\;\right.\left.,\;\cdots \;,\;T_{\psi }^{D}(1)\;\right\}$$

To compute the specific numerical values of the individual wavelet coefficients in Equation (2), we provide here the calculation process for the two-dimensional discrete wavelet vertical component coefficients in the *j*-th scale space, as described in the following equations:

(4) $$T_{\varphi }^{V}(j)=\frac{1}{\sqrt{NM}}\sum_{n=0}^{N-1}\sum_{m=0}^{M-1}T_{\varphi }^{}(j+1)\;\psi_{j}^{V}(n,m)$$

(5) $$\psi_{j}^{V}(n,m)=\psi_{j}(n)\;\varphi_{j}(m)$$

(6) $$\varphi_{j}(x)=2^{\frac{j}{2}}\varphi \left(2^{j}x-k\right)$$

(7) $$\psi_{j}(x)=2^{\frac{j}{2}}\psi \left(2^{j}x-k\right)$$

In these equations, $T_{\varphi }^{V}(j)$ represents the wavelet coefficients of the vertical component, M and N denote image dimensions, $T_{\varphi }^{}(j+1)$ signifies the input image from the previous level, $\psi_{j}^{V}(n,m)$ stands for the two-dimensional vertical component wavelet function, $\varphi_{j}(x)$ represents the one-dimensional scaling function, $\psi_{j}(x)$ denotes the one-dimensional wavelet function, j represents the scaling parameter, and k is the translation parameter. By scaling and translating the wavelet basis functions, a complete set of scaling functions and wavelet functions can be obtained. Likewise, one can calculate the wavelet coefficients $T_{\varphi }^{A}(j)$ on the approximation component, the wavelet coefficients $T_{\varphi }^{H}(j)$ on the horizontal component, and the wavelet coefficients $T_{\varphi }^{D}(j)$ on the diagonal component.

This process necessitates the determination of two parameters: the wavelet basis function and the decomposition level j. The specific values for these parameters will be provided in the experimental section.

### 3.3. Stripe Removal

**Figure 3:**
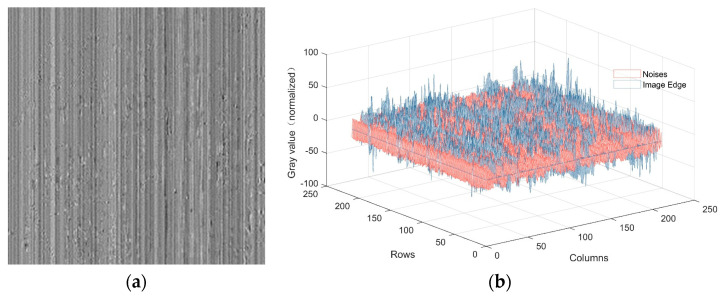
Vertical component in wavelet domain: (**a**) Two-dimensional; (**b**) Three-dimensional.

The primary cause of stripe in infrared images is the inconsistency in amplification parameters among the columns of the imaging device. This noise predominantly manifests in the form of columnar or horizontal striped noise within the image. Furthermore, this noise exhibits characteristics of global stationarity and subtle amplitude variations in the spatial domain. A significant advantage of wavelet decomposition lies in its directional sensitivity, as depicted in Figure 3, and post wavelet decomposition, as the striped noise is primarily concentrated in the vertical component. This paper assumes that all instances of stripe are situated within the vertical component, thereby minimizing potential disruptions to the intricate details present in the original image.

Figure 3b depicts the spatial three-dimensional distribution of this vertical component. It can be observed that the vertical component includes not only globally flat regions with evident directionality but also local sharp information. To reduce the influence of residual image details on noise estimation, we attempt to separate the image detail information from the vertical component. The simplest approach is to use wavelet thresholding segmentation [31], where values greater than the threshold are retained, while values below the threshold are set to zero. This approach can somewhat extract stripe noise, but the choice of threshold often exhibits significant fluctuations with changing scenes, leading to unstable segmentation results.

This paper proposes the utilization of the K-means clustering algorithm to adaptively segregate the stripe based on the scene context. Initially, the entities to be separated consist of grayscale values of different intensities, independent of their spatial distribution. Consequently, the two-dimensional wavelet domain’s vertical component is sequentially extracted by column, reducing it to a one-dimensional representation. This transformation facilitates the conversion of the problem into a one-dimensional clustering task, as shown in Equations (8) and (9).

(8) $$T_{\psi }^{V}(m,n)=\left[\begin{array}{ccccc} t_{1,1}^{v} & t_{1,2}^{v} & t_{1,3}^{v} & \cdots & t_{1,n}^{v}\\ t_{2,1}^{v} & t_{2,2}^{v} & t_{2,3}^{v} & \cdots & t_{2,n}^{v}\\ \vdots & \vdots & \vdots & \ddots & \vdots \\ t_{m,1}^{v} & t_{m,2}^{v} & t_{m,3}^{v} & \cdots & t_{m,n}^{v} \end{array}\right]$$

(9) $$T_{\psi }^{V}(1,m\times n)=reshape\;\left\{\;T_{\psi }^{V}(m,n)\;\right\}=\left[t_{1,1}^{v}t_{2,1}^{v}t_{3,1}^{v}\cdots t_{m-1,n}^{v}t_{m,n}^{v}\;\right]$$

where $T_{\psi }^{V}(m,n)$ denotes the vertical component of the wavelet domain of the degraded image, $t^{v}$ is the element in the component, and reshape denotes a descending transformation of the matrix read by columns.

Performing one-dimensional clustering analysis on the elements in set $T_{\psi }^{V}(1,m\times n)$, the process begins by selecting k elements from set $T_{\psi }^{V}(1,m\times n)$ to serve as initial cluster centers, denoted as μ. The distances between each sample $t_{}^{v}$ in set $T_{\psi }^{V}(1,m\times n)$ and each centroid $\mu_{i}$ are computed. Utilizing Equation (10), the clustering results for individual elements are determined, thus defining clusters $C_{t}$. Subsequently, recalculating centroids $\mu_{j}$ for all sample points in set $C_{t}$ follows, as depicted in Equation (11). This sequence of operations is iterated until all centroids cease to undergo changes, thereby producing the final classification outcome C. The clusters are then arranged based on the absolute values of their cluster centers, as described in Equation (12).

(10) $$E=\sum_{i=1}^{k}\sum_{t\in Ci}{\left\|t^{v}-\mu_{i}\right\|}_{2}^{2}$$

(11) $$\mu_{j}=\frac{1}{\left|C_{i}\right|}\sum_{t_{i,j}^{v}\in Ci}t_{i,j}^{v}$$

(12) $$T_{\psi }^{V}(1,m\times n)=\left\{\;C_{1}\;C_{2}\;\cdot \cdot \cdot \;C_{k}\;\right\}$$

In Equation (10), k represents the number of clusters to be formed, $C_{i}$ signifies the resulting clusters after clustering, and $t_{}^{v}$ represents the elements of the vertically reduced components. μ corresponds to the cluster centers of each cluster. For all subsequent iterations, apart from the initialization, the cluster centers μ are determined as the average of elements within each cluster. By iteratively minimizing Equation (10), the division of various clusters is achieved.

Upon the completion of clustering, the collection of clusters with relatively smaller absolute cluster centers is designated as stripe $T_{\psi }^{V,s}(m,n)$, while the collection of clusters with relatively larger absolute cluster centers is defined as residual image detail information $T_{\psi }^{V,r}(m,n)$, as illustrated in Equation (13).

(13) $$\begin{array}{cc} T_{\psi }^{V}(m,n) & =reshape\;\left\{\;C_{1}\;C_{2}\;0\;\cdot \cdot \cdot \;0\right\}+reshape\;\left\{\;0\;0\;C_{3}\;\cdot \cdot \cdot \;C_{k}\;\right\}\\ & =T_{\psi }^{V,s}(m,n)+T_{\psi }^{V,r}(m,n) \end{array}$$

**Figure 4:**
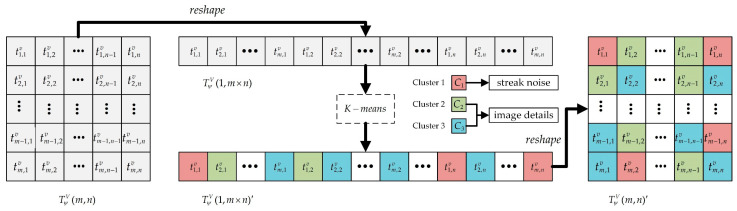
Framework of the striped noise separation.

At this point, we have completed the initial separation of the stripe non-uniformity noise, and the framework of the separation process is shown in Figure 4.

**Figure 5:**
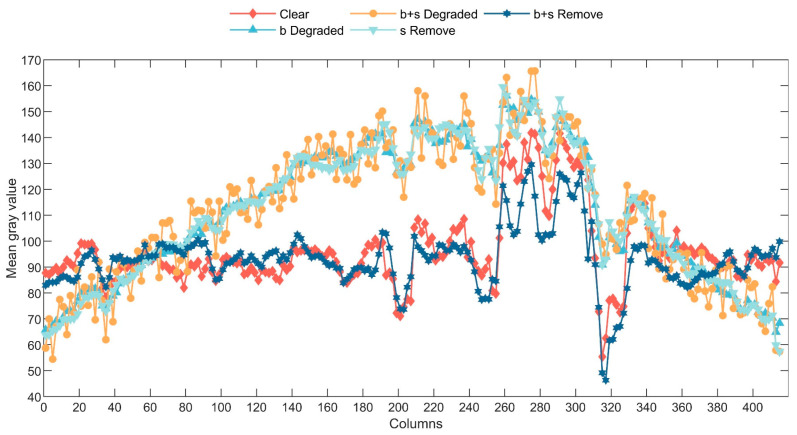
Column means of five sets of image gray values.

The aforementioned separated non-uniformity noise $T_{\psi }^{V,s}(m,n)$ does not directly represent the actual imaging device response deviation. However, its overall trend reflects the fundamental characteristics of true non-uniformity noise. To quantitatively compare the changing trend of this bias, Figure 5 illustrates the variations in the mean values of image columns. Evidently, the mean values of columns in the original image exhibit excessive smoothing. Following the introduction of striped noise, the neighboring column’s mean values experience distinct fluctuations, and the column mean of the image increases only locally after the introduction of thermal radiation noise, which shows a strong correlation between the fluctuations in the column means and the noise of streak-shaped inhomogeneity.

Consequently, it becomes plausible to employ the suppression of fluctuations in the mean values of columns within the vertical component to achieve the mitigation of striped noise. In this method, this corresponds to the suppression of the mean values of the separated striped noise image $T_{\psi }^{V,s}$ columns, with the specific calculation outlined as depicted in Equation (14):

(14) $$T_{\psi }^{V}(corrected)=T_{\psi }^{V}-\left[\frac{\sum_{i=1}^{M}T_{\left(i,1\right)}^{V,S}}{\sum_{i=1}^{M}\left(T_{\left(i,1\right)}^{V,S}\neq 0\right)}\frac{\sum_{i=1}^{M}T_{\left(i,2\right)}^{V,S}}{\sum_{i=1}^{M}\left(T_{\left(i,2\right)}^{V,S}\neq 0\right)}\cdots \frac{\sum_{i=1}^{M}T_{\left(i,N\right)}^{V,S}}{\sum_{i=1}^{M}\left(T_{\left(i,N\right)}^{V,S}\neq 0\right)}\right]$$

In Equation (14), $T_{\psi }^{V}(corrected)$ represents the corrected vertical component, and the parentheses encompass the calculation of the non-zero mean values of columns in $T_{\psi }^{V,s}(m,n)$. With this step, the correction of stripe within the wavelet domain vertical component concludes. Through the inverse wavelet transform, an image devoid of stripe can be obtained. By analyzing Figure 5, it becomes evident that the aforementioned process significantly suppresses fluctuations in the mean values of columns. The post-processed mean values of columns closely resemble those of the original image, and specific image-processing effects will be showcased in subsequent experimental stages.

### 3.4. Thermal Radiation Noise Removal

**Figure 6:**
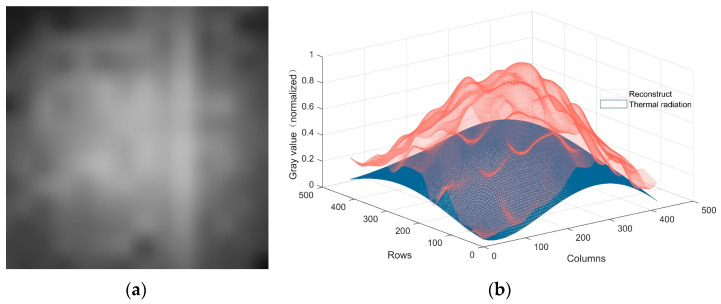
Reconstructed image with low-frequency components after wavelet transform: (**a**) 2D; (**b**) 3D.

Reconstructing a new image using the low-frequency component of the wavelet domain transform can effectively remove all the high-frequency information of the image. As shown in Figure 6a, the reconstructed image mainly contains thermal radiation noise as well as low-frequency information of potentially clear images, as shown in Figure 6b.

Based on the global smoothness characteristics of the thermal radiation noise [32,33], this paper uses the Bezier surface fitting algorithm to estimate its approximation. The process of Bézier surface fitting is expressed as follows:

(15) $$b(u,v)=\sum_{i=0}^{m}\sum_{j=0}^{n}P_{i,j}^{}\times B_{i,m}^{}(u)B_{j,n}^{}(v)$$

(16) $$B_{i,n}(t)=C_{n}^{i}{\left(1-t\right)}^{n-i},t\in [0,1]$$

where m and n signify the degrees of the surface, and $P_{i,j}^{}$ represents the coordinates of the Bézier surface control points. $B_{i,n}^{}(t)$ designates the Bernstein polynomial. The aforementioned system of equations can be transmuted into a matrix equation representation as follows:

(17) $$\left[\begin{array}{cccc} B_{0,m}\left(u_{1}\right)B_{0,n}\left(v_{1}\right) & B_{0,m}\left(u_{1}\right)B_{1,n}\left(v_{1}\right) & \cdots & B_{m,m}\left(u_{1}\right)B_{n,n}\left(v_{1}\right)\\ B_{0,m}\left(u_{2}\right)B_{0,n}\left(v_{2}\right) & B_{0,m}\left(u_{2}\right)B_{1,n}\left(v_{2}\right) & \cdots & B_{m,m}\left(u_{2}\right)B_{n,n}\left(v_{2}\right)\\ \vdots & \vdots & \ddots & \vdots \\ B_{0,m}\left(u_{r,s}\right)B_{0,n}\left(v_{r,s}\right) & B_{0,m}\left(u_{r,s}\right)B_{1,n}\left(v_{r,s}\right) & \cdots & B_{m,m}\left(u_{r,s}\right)B_{n,n}\left(v_{r,s}\right) \end{array}\right]\left[\begin{array}{c} P_{\left(0,0\right)}\\ P_{\left(0,1\right)}\\ \vdots \\ P_{\left(m,n\right)} \end{array}\right]=b$$

**Figure 7:**
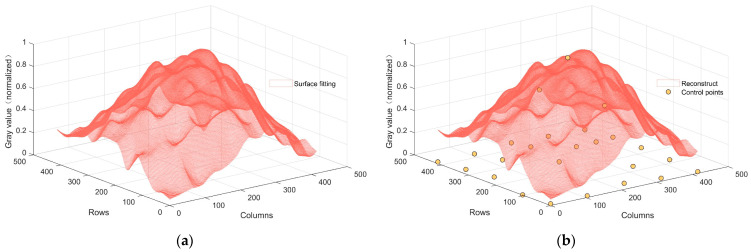
Schematic representation of Bezier surface control points: (**a**) 3D reconstructed image; (**b**) 3D Bezier surface control points.

The pivotal steps of Bezier surface fitting reside in the judicious selection of control points, wherein we utilize Equation (17) for the inverse determination of a set of control point coordinates. The sparsity matrix B within Equation (17) can be directly computed and acquired. Therefore, by defining the post-wavelet-transform low-frequency image as the Bezier surface b within Equation (17), it becomes immediately feasible to ascertain a set of requisite control points through the process of solving Equation (17) within the realm of least squares. The solved control points are shown in Figure 7.

Because the control point computation process employs an approximation of the low-frequency image as the fitted Bezier surface, the fitted surface not only incorporates thermal radiation noise but also encompasses the overall low-frequency information of the image. Directly subtracting the surface controlled by these control points for non-uniformity correction results in an issue of excessive darkness within the image scene due to this approach. In order to harmonize the luminosity information of the post-correction image and attain a judicious thermal radiation noise parameter b, this manuscript employs a coefficient λ to attenuate the noise image $\tilde{b}$ generated through the resolution of control points, as illustrated in Equation (18). The coefficient λ is realized by minimizing the energy function.

(18) $$\;b=\lambda \times \tilde{b}$$

(19) $$\;\lambda =\arg\min\left[J(\lambda )\right]$$

(20) $$J(\lambda )=\left\{std\left[mean(I-\lambda b)\right]-grad(I-\lambda b)\right\}$$

In Equation (20), mean denotes the computation of the average value for each column of the image, yielding a one-dimensional array. Meanwhile, std signifies the calculation of the standard deviation for this array. The term grad pertains to the derivation of the image’s gradient. Within the context of this study, the Sobel operator is employed to compute the first-order gradient of the image.

**Figure 8:**
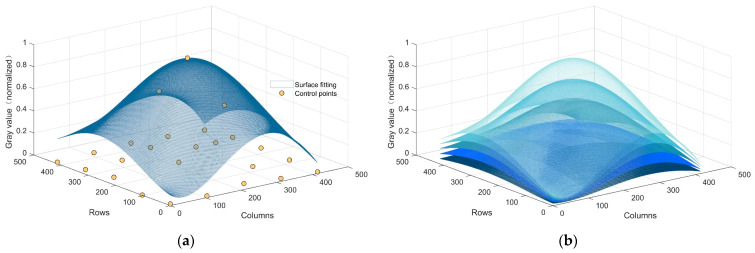
Optimal thermal radiation noise search process: (**a**) original thermal radiation noise; (**b**) thermal radiation noise under different degrees of attenuation. The light blue color at the top uses a coefficient λ of 1, the dark blue color at the bottom uses a coefficient λ of 0.2, and in the middle, from lighter to darker, the coefficients used are 0.8, 0.6, and 0.4 in that order.

Within the energy function J(λ), the first term corresponds to the level of uniformity across the image. A smaller value for this term signifies a higher degree of overall image uniformity. The second term represents the fidelity of gradients within the post-corrected image. This term is employed to mitigate issues such as contrast reduction and excessive dimness caused by the excessive attenuation of low-frequency components. The noise intensity coefficient λ can be dynamically adjusted based on the magnitude of thermal radiation noise, thus facilitating the regulation of image luminosity. In order to solve the energy function, a direct search method with a step size of 0.1 within the range of [0–1] is employed to acquire the optimal thermal radiation intensity coefficient. Subsequently, this parameter is applied to Equation (18), yielding the corrected image. The thermal radiation noise images obtained using coefficients λ of varying magnitudes are depicted in Figure 8.

## 4. Experimental Results

In this section, we evaluate the performance of the proposed method and compare it with the methods proposed by Zhang et al. [22], Li et al. [20], Cao et al. [21], Li-F et al. [23], Shi et al. [25], and Hong et al. [26]. All seven methods, including ours, are capable of achieving non-uniformity correction based on single-frame images. For our experiments, the non-uniformity correction for the simulated images was performed using the NWPU VHR-10 dataset [34,35,36], and the addition of thermal radiation noise was referenced to the degradation model of Shi et al. [25]. The BU-TIV dataset [37] was used for the non-uniformity correction of the real images. The parameters that need to be predetermined within the method are the number of wavelet transform iterations, denoted as j, and the number of clusters in the k-means clustering, denoted as k. Upon validation through experimental outcomes, we establish the parameter values as j=5 and k=4 and use sym5 as the wavelet basis function for this experiment.

### 4.1. Simulated Image Correction

To validate the performance of the proposed correction method, we employ PSNR, MSE, and SSIM to compare the denoising efficacy of the seven contrasting methods. The PSNR between two images x and y of size m×n is calculated as

(21) $$PSNR(x,y)=10\cdot \log_{10}\left(\frac{MAX_{x}^{2}}{\mathrm{MSE}(x,y)}\right)$$

where MAX is the maximum value that represents the grayscale of an image, which is 255 if the sampling point is represented by 8 bits. MSE denotes the mean square error, and for two grayscale images x and y of dimensions m×n, the mean squared error is defined as

(22) $$MSE(x,y)=\frac{1}{mn}\overset{m-1}{\underset{i=0}{\sum }}\overset{n-1}{\underset{j=0}{\sum }}{\left[x\left(i,\;j\right)-y(i,j)\right]}^{2}$$

**Figure 9:**
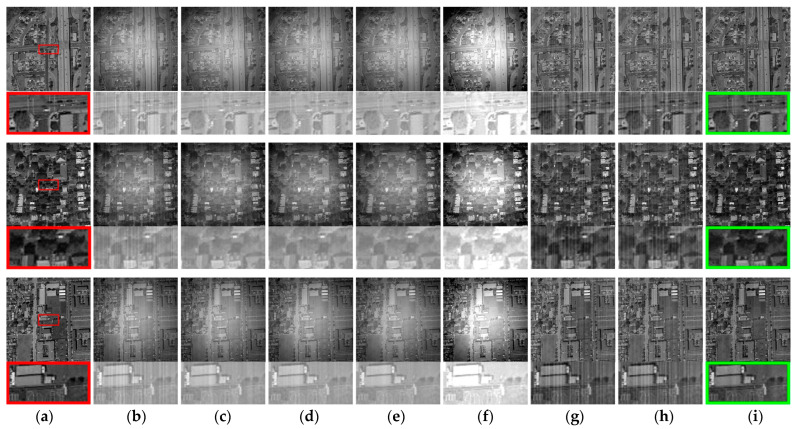
Comparative analysis of correction efficacy across seven distinct methods on simulated images: (**a**) clear image; (**b**) degraded image; (**c**) Zhang et al. [22]; (**d**) Li et al. [20]; (**e**) Cao et al. [21]; (**f**) Li-F et al. [23]; (**g**) Shi et al. [25]; (**h**) Hong et al. [26]; (**i**) proposed. The red box in the figure indicates a localized zoomed-in view of the selected area, and the green box labels the method proposed in this paper.

The *SSIM* between two images x and y of size m×n is calculated as

(23) $$SSIM(x,y)=\frac{\left(2\mu_{x}\mu_{y}+C_{1}\right)\left(2\sigma_{xy}+C_{2}\right)}{\left(\mu_{x}^{2}+\mu_{y}^{2}+C_{1}\right)\left(\sigma_{x}^{2}+\sigma_{y}^{2}+C_{2}\right)}$$

where $\mu_{x}$, $\mu_{y}$, $\sigma_{x}^{}$, $\sigma_{y}^{}$, and $\sigma_{xy}$, respectively, stand for the local mean, standard deviation, and covariance of the image. The correction outcomes from the three sets of simulation experiments are illustrated in Figure 9 and Table 1.

The simulated experiments demonstrate that Zhang’s, Li’s, Cao’s, and Li-F’s methods perform well in removing stripe non-uniformity noise, and the most effective of these is Cao’s method. However, when confronted with the superimposed global thermal radiation non-uniformity noise, they struggle to eliminate the residual non-uniformity stripes completely, resulting in lingering non-uniformity stripe noise in detail regions. Furthermore, there remains an evident global luminosity non-uniformity, as illustrated in Figure 9c–f, and LI-F’s method even amplifies the original thermal radiation noise. While this enhancement leads to improved SSIM scores, the improvement in PSNR metrics is not significant. Shi’s and Hong’s methods exhibit distinct efficacy in the removal of thermal radiation non-uniformity noise. However, the corrected images from these methods still contain conspicuous remnants of stripe non-uniformity noise, as illustrated in Figure 9g,h, thereby impacting the SSIM scores. In contrast, the proposed method in this paper effectively addresses both stripe non-uniformity noise and thermal radiation non-uniformity noise, yielding images with minimal residual non-uniformity noise. This results in significant improvements in both PSNR and SSIM metrics. In addition, the MSE index is more effective in responding to the variability of all pixels before and after correction, and the algorithm proposed in this paper has the smallest MSE among the seven methods, which demonstrates the excellent recovery performance of this algorithm.

Additionally, the introduction of a noise intensity parameter in the process of thermal radiation non-uniformity removal in this paper allows for adaptive adjustments based on the thermal radiation noise characteristics. This facilitates an improved preservation of image contrast, causing the corrected image to more closely resemble the original image. In contrast, the methods by Shi and Hong tend to yield corrected images that are globally brighter.

### 4.2. Real Image Correction

**Figure 10:**
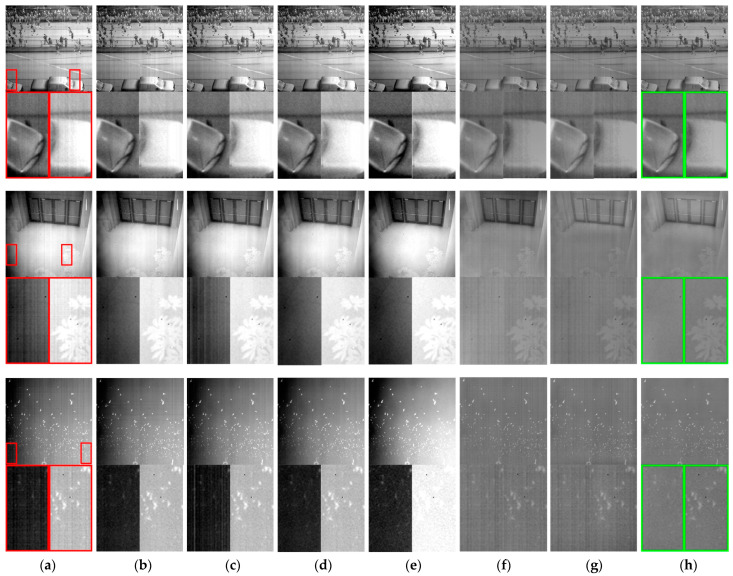
Comparative analysis of correction efficacy across seven distinct methods on real images: (**a**) degraded image; (**b**) Zhang et al. [22]; (**c**) Li et al. [20]; (**d**) Cao et al. [21]; (**e**) Li-F et al. [23]; (**f**) Shi et al. [25]; (**g**) Hong et al. [26]; (**h**) proposed. The red box in the figure indicates a localized zoomed-in view of the selected area, and the green box labels the method proposed in this paper.

**Figure 11:**
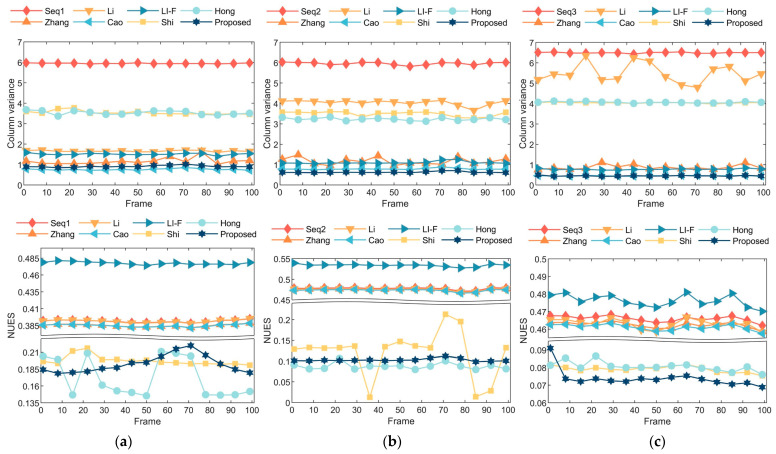
Performance evaluation of five methods across three distinct real-world scenarios: (**a**) Scenario 1—street; (**b**) Scenario 2—indoors; (**c**) Scenario 3—skyscape.

In this section, three sets of real infrared image sequences containing authentic non-uniformity noise are employed. Figure 10 displays the correction outcomes achieved by these seven methods on the three image sequences. To conduct a further objective performance comparison of different correction methods, the column variance index $Var_{c}$ is employed to evaluate stripe non-uniformity noise, while the national standard non-uniformity index NUES is used for thermal radiation non-uniformity noise [38]. Figure 11 presents the correction outcome metrics for the three image sequences. 

The column variance $Var_{c}$ for evaluating stripe non-uniformity noise is defined as

(24) $$Var_{c}=\frac{1}{N-1}\sum_{j=1}^{N-1}{\left(I_{j}^{d}-{\bar{I}}^{d}\right)}^{2}$$

(25) $$I^{d}=\left[\sum_{i=1}^{M}I_{i,2}\sum_{i=1}^{M}I_{i,3}\cdots \sum_{i=1}^{M}I_{i,N}\right]-\left[\sum_{i=1}^{M}I_{i,1}\sum_{i=1}^{M}I_{i,2}\cdots \sum_{i=1}^{M}I_{i,N-1}\right]$$

(26) $${\bar{I}}^{d}=\frac{1}{N}\sum_{j=1}^{N-1}I_{j}^{d}$$

In these equations, $I^{d}$ represents the first-order difference of the column means, reflecting the changes in grayscale values between adjacent columns in the image. The column variance metric assesses the non-uniformity of image gains and biases across different channels. A smaller value of this metric indicates a higher degree of image uniformity.

NUES is defined as follows:

(27) $$NUES=\frac{1}{\bar{I}}\sqrt{\frac{1}{M\cdot N-d-h}\sum_{i=1}^{M}\sum_{j=1}^{N}{\left(I_{i,j}-\bar{I}\right)}^{2}}$$

(28) $$\bar{I}=\sum_{i=1}^{M}\sum_{j=1}^{N}I_{i,j}/(M\cdot N)$$

In these equations, $I_{i,j}$ represents the grayscale value at the pixel position (i,j), and $\bar{I}$ signifies the global average grayscale value of the image. The image non-uniformity index reflects the degree of uniformity in the overall grayscale values of the image. In the computed results, a smaller value of NUES indicates a lower level of non-uniformity noise in the image.

Three sets of image sequences were each processed with 100 frames for correction. The results depicted in Figure 10 show the outcomes of processing the first frame of each sequence. It is evident that the performance of the proposed method in this paper surpasses that of the comparative methods. The correction methods by Zhang, Li, Cao, and Li-F exhibit unsatisfactory performance in removing thermal radiation non-uniformity noise, leading to pronounced global brightness non-uniformity in the images. Notably, Li’s method results in a ghosting phenomenon in localized regions of the noisy image, as illustrated in Figure 10c. LI-F’s method causes part of the effective information to be submerged in the image background due to the amplified thermal radiation noise, as shown in Figure 10e. The methods by Shi and Hong are effective in ameliorating the presence of thermal radiation non-uniformity noise in images. However, they struggle to effectively suppress high-frequency stripe noise, as evident in the corrected images shown in Figure 10f,g, where residual stripe noise remains conspicuous.

In contrast, the proposed method in this paper renders high-frequency non-uniformity noise virtually imperceptible, significantly curtails low-frequency thermal radiation non-uniformity noise, and, upon visual inspection, yields the clearest corrected images with minimal apparent residual noise.

In Figure 11, the trends in the statistical metrics align with the expected processing outcomes. Specifically, the methods proposed by Zhang, Cao, and Li-F exhibit notable suppression effects on high-frequency stripe non-uniformity noise, as illustrated in the first row of Figure 11. Cao’s method closely approximates the performance of the proposed method in this paper. Li’s method performs well in Scenario 1 but exhibits poor performance with notable fluctuations in Scenarios 2 and 3. This fluctuation is attributed to the presence of significant vignetting in Scenarios 2 and 3, where Li’s method tends to generate ghosting artifacts in dark corner regions. Shi’s and Hong’s methods display distinct efficacy in suppressing low-frequency thermal radiation non-uniformity noise, as shown in the second row of Figure 11. The results from these two methods are comparable to the proposed method in this paper, with Hong’s method slightly outperforming the proposed method in Scenario 2. However, Hong’s method exhibits pronounced fluctuation in correction efficacy across different image scenarios, whereas the proposed method demonstrates stable correction performance across diverse scenarios.

In contrast to the comparative methods, which excel in addressing specific types of noise but significantly compromise correction efficacy when handling combined noise, the proposed method in this paper effectively achieves the joint correction of both high-frequency and low-frequency non-uniformities.

Table 2 presents the average column variance index $Var_{c}$ for 100 frames of each image sequence. Table 3 provides statistics on the average non-uniformity index NUES for 100 frames of each image sequence. Lower values in both tables indicate better image consistency. From Table 2, it is evident that the proposed method yields the lowest column variance index for all three image sequences after correction. Table 3 highlights that the proposed method significantly enhances the non-uniformity index for all three image sequences after correction while maintaining a stable correction performance. The average non-uniformity index for the three image sequences decreases by 75.54%.

Table 4 provides statistics on the average processing time for one frame of each image sequence using the seven processing methods. From the table, it is evident that the methods by Zhang, Li, Cao, and Li-F outperform the others in terms of processing time. Shi’s and Hong’s methods exhibit significantly longer processing times, primarily due to the substantial computational effort required to solve the optimal function when estimating the thermal radiation noise. The processing time of the proposed method in this paper is slightly higher than that of the first four methods. This can be attributed to the clustering method involved in this paper’s approach, which incurs noticeable increases in processing time with changing input image scales.

By considering the results from Table 2, Table 3 and Table 4 collectively, it becomes apparent that the proposed method in this paper outperforms the comparative methods in terms of image correction efficacy, with processing times comparable to those of similar correction methods. Consequently, the proposed method is better suited to applications demanding both real-time image quality and processing speed.

**Figure 12:**
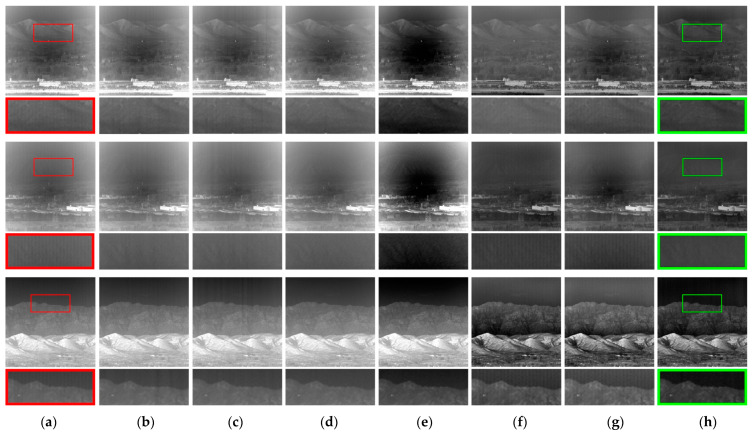
Comparative analysis of correction efficacy across seven distinct methods on images from a wider range of scenes: (**a**) degraded image; (**b**) Zhang et al. [22]; (**c**) Li et al. [20]; (**d**) Cao et al. [21]; (**e**) Li-F et al. [23]; (**f**) Shi et al. [25]; (**g**) Hong et al. [26]; (**h**) proposed. The red box in the figure indicates a localized zoomed-in view of the selected area, and the green box labels the method proposed in this paper.

To further illustrate the effectiveness of the algorithm proposed in this paper, Figure 12 shows more examples of corrections based on real noisy images [39].

## 5. Discussion

To surmount the challenges posed by various adverse conditions, such as insufficient scene motion and the amalgamation of high-frequency and low-frequency noise, we have proposed a single-frame image non-uniformity correction algorithm grounded in wavelet domain noise separation. This approach obviates the need for calibration experiments, enabling the simultaneous removal of both stripe noise and thermal radiation noise from images. Its capacity to execute correction with a sole image implies operability even in entirely static scenes. Comparative experiments were conducted against six representative single-frame image non-uniformity correction algorithms, yielding exceptional results.

### 5.1. Interpretation of Results

In the experimental section, as outlined in Section 4, it was observed that the methods proposed by Zhang, Li, Cao, and Lif effectively eliminate high-frequency stripe noise while exhibiting limited effectiveness in removing low-frequency noise, as depicted in Figure 9c–i, along with Figure 10 and Figure 11, which share a similar trend. Among them, Cao’s method demonstrated the most favorable performance. The approach presented in this paper exhibits similar high-frequency noise removal efficacy to Cao’s approach. On the other hand, the methods developed by Shi and Hong exhibit proficiency in removing low-frequency thermal radiation noise while showing limited effectiveness in high-frequency noise removal. Both exhibit similar denoising capabilities, yet these two methods differ significantly in terms of processing speed. As revealed in Table 4, Hong’s method incurs the longest computational time, primarily due to its employment of progressive iterative parameter updates to enhance fitting precision. The approach presented in this paper aligns with the subjective visual results of these two methods in terms of low-frequency noise removal, as evident in Figure 9g–i, along with Figure 10 and Figure 11. Notably, the proposed method results in corrected images that closely approximate the overall brightness of the original reference image. This is primarily attributed to the introduction of a noise intensity parameter during the thermal radiation non-uniformity elimination process in this paper, enabling adaptive adjustments based on thermal radiation noise characteristics. In contrast, the methods by Shi and Hong tend to produce overall brighter correction images.

The experimental results affirm the efficacy of our approach in concurrently addressing both stripe noise and thermal radiation noise. This effectiveness is chiefly ascribed to the deployment of wavelet transform for the differentiation of various noise types within our scheme. Denoising is accomplished through the utilization of distinct modules for stripe noise extraction and thermal radiation fitting, distinguishing our approach from the typical focus on the removal of a singular type of noise. It is imperative to emphasize that our proposed scheme is not a mere amalgamation of existing approaches but represents a novel solution for distinct noise types. Table 4 serves as a corroboration of this assertion, demonstrating that our proposed scheme achieves both low-frequency and high-frequency noise reduction without a significant escalation in computational time.

### 5.2. Limitations

The single-frame image-based non-uniformity correction algorithm that we have proposed yields satisfactory results in most scenarios. However, our approach remains subject to certain limitations.

**Figure 13:**
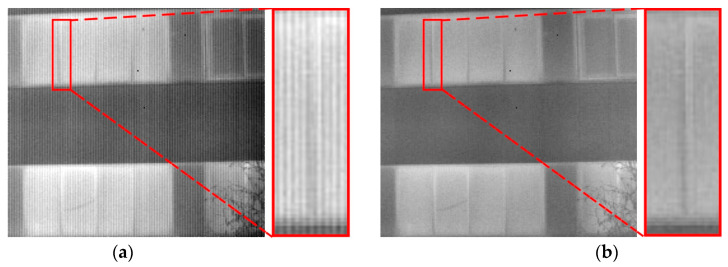
Failure case of the proposed non-uniformity correction method: (**a**) input noisy image; (**b**) non-uniformity correction result provided using our method. The red box in the figure indicates a localized zoomed-in view of the selected area.

Figure 13 illustrates a case in which our proposed algorithm fails. In scenarios involving image edge details with structures and grayscale values resembling non-uniformity noise, our method may make incorrect assessments. As shown within the rectangular box in Figure 13a, in this localized area, there is a window’s frame edge. After processing with our algorithm, the frame’s width reduces from six pixels, as shown in Figure 13a, to three pixels, as seen in Figure 13b. The reason for this discrepancy lies in the fact that the detailed information is vertically distributed and has an overall width of only a few pixels. Upon closer examination of the enlarged image, it becomes apparent that this pertinent information closely resembles non-uniformity noise. Wavelet transformation and clustering algorithms are unable to accurately separate the Striped non-uniformity noise from this valuable information, leading to a misjudgment of the non-uniformity noise. In future endeavors, we will propose an enhancement algorithm based on prior information from the original image’s detail to further enhance the accuracy of non-uniformity correction.

## 6. Conclusions

In this paper, we propose a method for the correction of non-uniformity in single-frame infrared images based on noise separation in the wavelet domain. More specifically, we commence by decomposing the noisy image into distinct frequency components through wavelet transformation. Subsequently, we employ a clustering algorithm to extract high-frequency noise from the vertical components within the wavelet domain, concurrently employing a method of surface fitting to capture low-frequency noise from the approximate components within the wavelet domain. This approach, devoid of inter-frame correlation dependencies, ensures the efficacious removal of stripe noise and thermal radiation noise, simultaneously mitigating the issue of ghosting artifacts. Experimental outcomes corroborate that, when compared with other cutting-edge single-frame non-uniformity correction techniques, our proposed methodology excels in the reduction of superimposed noise while preserving the fidelity of post-correction image details, with no conspicuous issues regarding ghosting artifacts. Moreover, our algorithm does not impose significant computational overhead in the elimination of superimposed noise, thereby underscoring the practicality of the proposed approach, especially in applications demanding both image quality and processing speed. In future research endeavors, our emphasis will pivot towards the development of real-time non-uniformity correction algorithms tailored for engineering applications.

## Figures and Tables

**Table 1:** Comparative assessment of the performance of seven methods on simulated images.

Image	Index	Degraded	Zhang. [22]	Li. [20]	Cao. [21]	Li-F. [23]	Shi. [25]	Hong. [26]	Proposed
Street	MSE	1475	1483	1445	1492	3989	335.62	324.08	162.25
	PSNR	16.44	16.49	16.53	16.39	12.12	22.87	23.02	26.03
	SSIM	0.71	0.80	0.81	0.77	0.80	0.74	0.75	0.94
Residence	MSE	1917	1877	1887	1894	5069	339.25	339.48	234.70
	PSNR	15.31	15.39	15.37	15.36	11.08	22.83	22.18	24.43
	SSIM	0.66	0.76	0.76	0.77	0.76	0.71	0.71	0.93
Plants	MSE	1737	1722	1712	1752	4373	373.94	422.04	180.27
	PSNR	15.73	15.76	15.79	15.70	11.72	22.40	21.88	25.57
	SSIM	0.70	0.78	0.79	0.77	0.81	0.74	0.74	0.93

**Table 2:** Assessment of roughness metrics for the seven methods.

Image	Index	Input	Zhang. [22]	Li. [20]	Cao. [21]	Li-F. [23]	Shi. [25]	Hong. [26]	Proposed
Scenario 1	$Var_{c}$	5.946	1.166	1.658	0.762	1.517	3.548	3.579	0.914
Scenario 2	$Var_{c}$	5.954	1.156	4.019	0.789	1.098	3.504	3.221	0.629
Scenario 3	$Var_{c}$	6.491	0.872	5.295	0.451	0.771	4.021	4.055	0.444

**Table 3:** Assessment of NUES metrics for the seven methods.

Frames 1–100	Index	Input	Zhang. [22]	Li. [20]	Cao. [21]	Li-F. [23]	Shi. [25]	Hong. [26]	Proposed
Scenario 1	NUES	0.391	0.385	0.391	0.384	0.478	0.098	0.179	0.142
Scenario 2	NUES	0.477	0.475	0.475	0.473	0.533	0.119	0.087	0.102
Scenario 3	NUES	0.466	0.462	0.463	0.461	0.476	0.079	0.081	0.073

**Table 4:** Evaluation of average time consumption for the seven methods.

Frames	Index	Zhang. [22]	Li. [20]	Cao. [21]	Li-F. [23]	Shi. [25]	Hong. [26]	Proposed
Scenario 1	Time/s	0.425	0.211	0.328	0.344	12.870	22.795	1.064
Scenario 2	Time/s	0.432	0.214	0.3162	0.382	6.472	65.664	1.049
Scenario 3	Time/s	0.428	0.222	0.3326	0.353	6.591	29.548	1.038

## Data Availability

Not applicable.
